# Prevalence and Correlates of Client-Perpetrated Violence against Female Sex Workers in 13 Mexican Cities

**DOI:** 10.1371/journal.pone.0143317

**Published:** 2015-11-23

**Authors:** Shirley J. Semple, Jamila K. Stockman, Eileen V. Pitpitan, Steffanie A. Strathdee, Claudia V. Chavarin, Doroteo V. Mendoza, Gregory A. Aarons, Thomas L. Patterson

**Affiliations:** 1 Department of Psychiatry, University of California San Diego, La Jolla, California, 92093–0680, United States of America; 2 Division of Global Public Health, Department of Medicine, University of California San Diego, La Jolla, California, 92093–0507, United States of America; 3 Evaluation and Research Department, Mexican Foundation for Family Planning (Mexfam), Distrito Federal, 14000, Mexico; David Geffen School of Medicine at UCLA, UNITED STATES

## Abstract

**Background:**

Globally, client-perpetrated violence against female sex workers (FSWs) has been associated with multiple health-related harms, including high-risk sexual behavior and increased exposure to HIV/STIs. This study examined correlates of client-perpetrated sexual, physical, and economic violence (e.g., robbery) against FSWs in 13 cities throughout Mexico.

**Methods:**

FSWs (N = 1,089) who were enrolled in a brief, evidence-based, sexual risk reduction intervention for FSWs *(Mujer Segura)* were interviewed about their work context, including experiences of violence perpetrated by clients, sexual risk and substance use practices, financial need, and social supports. Three broad categories of factors (sociodemographic, work context, behavioral and social characteristics of FSWs) were examined as correlates of sexual, physical, and economic violence.

**Results:**

The prevalence of different types of client-perpetrated violence against FSWs in the past 6 months was: sexual (11.7%), physical (11.8%), economic (16.9%), and any violence (22.6%). Greater financial need, self-identification as a street worker, and lower perceived emotional support were independently associated with all three types of violence. Alcohol use before or during sex with clients in the past month was associated with physical and sexual violence. Using drugs before or during sex with clients, injection drug use in the past month, and population size of city were associated with sexual violence only, and FSWs’ alcohol use score (AUDIT-C) was associated with economic violence only.

**Conclusions:**

Correlates of client-perpetrated violence encompassed sociodemographic, work context, and behavioral and social factors, suggesting that approaches to violence prevention for FSWs must be multi-dimensional. Prevention could involve teaching FSWs strategies for risk avoidance in the workplace (e.g., avoiding use of alcohol with clients), enhancement of FSWs’ community-based supports, development of interventions that deliver an anti-violence curriculum to clients, and programs to address FSWs’ financial need by increasing their economic opportunities outside of the sex trade.

## Introduction

Female sex workers (FSWs) are often exposed to work-related traumas in the form of sexual, physical, and economic violence (e.g., robbery, refusal to pay) perpetrated by clients [1–3]. Physical and sexual violence against FSWs has been associated with high-risk sexual behavior (e.g., inconsistent condom use) [4–7], increased risk for contracting HIV and other STIs [4,8,9], high levels of anxiety and depression [10–13], and post-traumatic stress disorder (PTSD) [14,15], as well as suicidal thoughts and attempts [16,17].

Violence against FSWs is pervasive in both developed countries and lower-to-middle-income countries (LMIC) [1]. In the US, Canada, Britain, and Australia, studies on violence against FSWs report that 40% to 82% of FSWs have ever been physically or sexually assaulted by a client [15,18–20]. Studies in LMIC have yielded similarly high prevalence estimates of client-perpetrated physical and sexual violence, ranging from 55% to 77% [7,21,22]. In Southern India, 26.4% of FSWs reported physical or sexual violence in the past year [4], and 9.6% reported experiencing client violence in the past six months [6]. In a study of brothel-based FSWs in Abuja, Nigeria, 52.5% had experienced any violence in the past six months [2], including sexual and physical violence (41.9% and 35.7%, respectively). In Kenya, one-third of sex workers reported sexual violence in the past year [23], and in Thailand, 14.6% of FSWs experienced client-perpetrated physical or sexual violence in the week prior to the survey [5].

In our previous work conducted with FSWs in two Mexican border cities (Tijuana & Ciudad Juarez), the prevalence of client-perpetrated abuse (physical, sexual, or emotional) in the past six months was found to be 31% [13].

Less is known about the prevalence of economic violence against FSWs. In Nigeria, 37.7% of brothel-based FSWs reported economic violence (e.g., defined as “exploitation”) by clients [2]. In Hong Kong, 18.6% reported refusal of payment by clients, and 14.2% reported being robbed [3]. Overall, 64% of FSWs reported experiencing economic violence in the form of robbery, theft (e.g., stolen cash), or refusal to pay for services [3]. In Uganda, economic violence against FSWs was common and involved their receiving less pay than negotiated (72%) or no pay at all from a client (56%) [24].

From the perspective of HIV/STI prevention, physical and sexual violence compromises FSWs’ ability to negotiate condom use and increases HIV/STI risk [9,21,25–29]. Physical trauma to the genital tract is believed to facilitate HIV/STI acquisition, especially among young girls [5]. Moreover, as suggested by others, male clients who perpetrate physical and sexual violence against FSWs may have higher rates of STIs, resulting in even greater risk for FSWs [5]. Structural and community mobilization interventions to reduce violence against FSWs are likely to increase access to HIV/STI prevention and treatment services [4], and thus slow the spread of these infectious diseases.

A variety of risk factors associated with client-perpetrated physical and sexual violence against FSWs have been identified. *Socio-economic characteristics* of FSWs that are risk factors for sexual violence include low income, low levels of education, a marital status of either cohabiting or having a primary partner [4,30], and younger age [4,31,32]. Financial need also makes FSWs more vulnerable to violence. In one study in India, physical violence against FSWs was associated with FSWs’ self-reported debt, an indicator of economic hardship [33]. In a study conducted in Tijuana, Mexico, unstable housing predicted work-related violence against FSWs [34].

FSWs’ *work context* also influences their risk for experiencing violence. Those who have a higher volume of clients are more vulnerable to physical, sexual, and economic violence [4,13,35]. FSWs who work in riskier settings (e.g., streets, alleys) have increased risk for experiencing client violence [20,34,36]. Substance use with clients represents another work-related factor associated with violence. This finding has been reported in both developed countries and LMIC, including Mexico, where client-perpetrated abuse has been associated with FSWs’ use of drugs with clients [13,37,38].

A number of FSWs’ *behavioral and social characteristics* have also been linked to client-perpetrated violence against them. Studies in Kenya, Uganda, and China report an association between FSWs’ alcohol consumption and experiences of client-perpetrated sexual violence [23,39,40]. Also, FSWs’ use of illegal drugs has been associated with their experiences of client-perpetrated violence [8,41]. Several studies of injection drug users (IDUs), including our work with FSW-IDUs in Mexico, have yielded an association between injection drug use and greater odds of sexual or physical assault in the workplace [17,35,42]. Less attention has been paid to the role of social support in ameliorating the negative impact upon FSWs of client-perpetrated violence. In a qualitative study, Romero-Daza et al. [43] found that the absence of social support increased the association between client violence and FSWs’ use of illicit drugs. Other studies have identified client-perpetrated violence and limited social support as co-occurring stressors in the lives of FSWs [40,44].

The present study builds upon our previous research on client-perpetrated abuse among FSWs in the Mexico-US border region [13,42] by exploring client-perpetrated violence against FSWs beyond the border region in 13 cities throughout Mexico. The border regions studied in our previous work are different from other locations in Mexico because of their proximity to major US cities (San Diego, CA and El Paso, Texas) and their status as international drug trafficking routes into the US [45,46]. Expanding our work on client-perpetrated violence against FSWs to include non-border locations in Mexico may yield different prevalence estimates and correlates of violence that have greater external validity.

The present study seeks to identify prevalence and correlates of three types of client-perpetrated violence against FSWs: sexual, physical, and economic. Identification of correlates that are unique to the various types of violence has the potential to inform the development of evidence-based HIV/STI prevention programs and treatment services for this high-risk population. Moreover, effective strategies for the treatment of trauma may vary by the type of violence experienced. Because FSWs in Mexico share problems with those in other LMIC, including poverty, childhood abuse history, police abuse, high rates of STIs, and high rates of drug and alcohol use [47–52], the findings from this study are potentially applicable to other LMIC.

## Methods

### Participants and Setting

The sample consisted of 1,089 FSWs who were enrolled in a sexual risk reduction intervention in 13 cities throughout Mexico [53]. Eligible women were biologically female, at least 18 years of age, self-identified as a FSW, reported having traded sex for money, drugs, shelter or other material benefit in the previous 2 months, had unprotected vaginal or anal sex with a client at least once during the previous 2 months, and tested HIV-negative at baseline [53]. The latter criterion was imposed because HIV seroconversion was a study endpoint. Data were collected between June 2011 and December 2013.

All study procedures were approved by the Institutional Review Board of the University of California, San Diego (Project 090320) and by the Ethics Committee at Mexfam. All procedures were conducted in accordance with the Helsinki Declaration. Written informed consent was obtained from all participants.

### Recruitment

Time-location sampling was used to recruit approximately 84 participants in each of the 13 cities (range = 71 to 87). This approach was used successfully to recruit FSWs in our previous studies in Mexico [54,55]. Trained outreach workers compiled a map of bars, brothels, hotels and motels, shooting galleries, alleys, and street corners, which were used as the sampling frame to recruit prospective participants in each city. For this study, FSWs who were interested were referred to the local Mexican Foundation for Family Planning (Mexfam) clinic, where they were screened for eligibility. Eligible participants completed a 2-hour baseline session that included the consent procedure, HIV and STI screening, a face-to-face interview, and the *Mujer Segura* (Healthy Woman) sexual risk reduction counseling intervention or a time-equivalent, standard counseling comparison condition [53]. The interview and the HIV/STI testing were repeated at a six-month follow-up visit. The current analyses were based on data gathered through the baseline interview.

### Procedure

The baseline interview lasted approximately 30 minutes. As in our previous studies with Mexican FSWs, the interview was administered by trained outreach workers using computer-assisted personal interviewing (CAPI) (NOVA Software, MD, USA).

### Measures

Three broad categories of variables, selected on the basis of previous research with FSWs in LMIC, were examined in relation to each type of violence. They included sociodemographic factors, work context variables, and selected behavioral and social characteristics of the FSWs. Since the population size of the cities in which the study sites were located could affect such factors as community norms and tolerance of sex work, HIV prevalence, and levels of risk behavior, we included this factor in our analyses.

#### Violence against FSWs

Three types of client-perpetrated violence were assessed in the baseline interview. Sexual violence in the past six months was measured by 10 items (e.g., how often has a client: forced you to have sex against your will (raped you); forced you to suck or lick someone else; raped you with a physical object; raped you using a weapon (gun, knife); forced you to watch a sex act; forced you to have sex with someone else; forced you to masturbate someone else; masturbated you against your will; forced you to kiss or touch someone else; kissed or touch you against your will). Physical violence in the past six months was measured by 5 items (e.g., how often have you been: stabbed by a client; tortured by a client; threatened with murder by a client; choked or strangled by a client; kidnapped by a client). Economic violence in the past six months was measured by 2 items (e.g., how often have you been: robbed by a client; not paid by a client). Response categories for all scale items ranged from 1 (never) to 4 (very often). Three dichotomized variables were created to represent FSWs’ experiences of sexual, physical, and economic violence in the past six months (1 = yes, 0 = no).

#### Sociodemographics

Participants reported their age, marital status, and number of years of education. Marital status was coded using 6 categories (married, separated or filing for divorce, divorced but not remarried, widowed, never married, or common-law), and recoded as a dichotomous variable for the analyses (married or common law = 1, other marital status = 0). Financial need was assessed with the question, “How would you rate your current financial situation?” Response categories were: extremely good, good, neither good nor bad, bad, and extremely bad. Responses were recoded to create a dichotomous variable that captured the FSW’s financial situation (1 = bad/extremely bad, 0 = other).

#### Work context

*Type of Sex Work*: Participants were presented with a list of nine types of sex worker (barmaid, dance hostess, taxi girl, brothel worker, street worker, lover, call girl/escort, companion for parties and vacations, other) and asked to indicate which term best described them. Since street-based sex workers have a particularly high risk for experiencing client violence [20,34,36], sex worker type was dichotomized as street sex worker = 1, other type = 0. *Use of Alcohol or Drugs Before or During Sex with Clients*: Participants were asked how often in the past month they had used (1) alcohol or (2) drugs before or during sex with a client. Response categories (never, sometimes, often, always) were recoded yes/no to create two dichotomous variables. *Total Number of Male Clients in the Past Month*: Participants were asked to report or estimate this figure.

#### Behavioral and social characteristics

*Alcohol Use in the Past Month*: The AUDIT-C scale is a 3-item, reliable alcohol screening instrument [56–58] that includes the following questions and response categories: (1) How often do you have a drink containing alcohol? (never, monthly or less, 2–4 times a month, 2–3 times a week, 4 or more times a week); (2) How many standard drinks containing alcohol do you have on a typical day? (1 or 2, 3 or 4, 5 or 6, 7 to 9, 10 or more); (3) How often do you have six or more drinks on one occasion? (never, less than monthly, monthly, weekly, daily or almost daily). Summary scores ranged from 0 to 12. *Number of Drugs Used in the Past Month*: Participants were asked to report on their use of 13 drugs in the past month, including marijuana, cocaine, crack, ecstasy, methamphetamine, and heroin. Frequency of use was rated for each drug on a scale from 1 (*never*) to 6 (*every day*), and responses were recoded as 1 (does use) or 0 (does not use). A summary variable was created to indicate total number of drugs used in the past month. *Injected in the Past Month*: For each of the 13 drugs described above, participants were asked to report their method of administration, which included “injected” as a response category. Participants who reported injection of any drug or of vitamins in the past month were assigned 1 for injection, 0 for no injection. *Perceived Emotional Support* was measured using a 7-item scale developed by Pearlin et al. [59] that assesses the presence in the respondent’s life of family members and friends whom the respondent perceives as caring, trustworthy, supportive, and able to keep a confidence. Items in statement form (e.g., “The people close to you let you know they care about you”) are rated on a 4-point scale ranging from 1 (strongly disagree) to 4 (strongly agree). Mean scores were computed and used in these analyses.

To explore potential differences in correlates of violence by study site, a variable representing the population size of each study site’s city was created and used as a control variable in the analyses. Population size categories were: less than 100,000 (coded 1); 100,000 to 499,999 (coded 2); 500,000 to 1 million (coded 3); and greater than 1 million (coded 4). The four categories were collapsed to create a dichotomously-scored variable according to which cities with 500,000 or more inhabitants were coded 1 and those with fewer than 500,000 were coded 0. Population-size figures were based on 2010 census data published by the National System of Municipal Information, National Institute for Federalism and Municipal Development (http://www.snim.rami.gob.mx).

### Data Analyses

We conducted descriptive analyses on baseline data of past-six month prevalence of sexual, physical, and economic violence. For the dataset, see S1 Dataset. Multivariate logistic regression analyses were then conducted for each violence outcome: sexual, physical, and economic. Thirteen independent variables (IVs) of three different types were included in each logistic regression. They were: age, number of years of education, marital status, financial need (socio-demographic variables); street sex worker versus other, used alcohol with client in past month, used drugs with client in the past month, number of clients in the past month (work context variables); and AUDIT score, number of drugs used in the past month, injected drugs in the past month, and perceived emotional support (behavioral and social characteristics of FSWs). As noted, we also controlled for the population size of the cities where the study sites were located. Each dependent variable was coded (1 = experienced this type of violence in past 6 months) or (0 = did not experience this type of violence in past 6 months). Two variables with skewed distributions (total number of clients, number of drugs used in past month) were log 10 transformed for analytic purposes.

## Results

### Description of Sample

Ninety percent of the sample was born in Mexico. The average age and number of years of education were 32.9 (SD = 9.7, range 18–67) and 7.1 years (SD = 3.3, range 0–21), respectively. The majority of FSWs were never married or were living in a common-law relationship (49.8% and 19.8% respectively). Eighty-eight percent had at least one child, and the average number of children was 2.8 (SD = 1.5, range 1–11). FSWs were most likely to be living with children (67.0%), spouse (19.8%), or parents (18.7%). On average, FSWs had been employed in the sex trade for 6.3 years (SD = 6.8, range = 1–40 years). Prevalence of the types of client-perpetrated violence against FSWs in the *past six months* was: sexual violence (11.7%), physical violence (11.8%), economic violence (16.9%), and any violence (22.6%). Among FSWs who reported any violence in the past six months, 53% reported experiencing more than one type of violence. Compared to FSWs who did not experience client violence in the past 6 months, FSWs who experienced violence were significantly more likely to work on the street, use alcohol before or during sex with clients, use drugs before or during sex with clients, report greater financial need, use a larger number of drugs in the past month, inject drugs, and live in a municipality with a population greater than 500,000 (Table 1).

**Table 1:** Socio-demographic, work context, and behavioral and social characteristics of FSWs who experienced client-perpetrated violence compared with those who did not (n = 1089).

	Experienced client-perpetrated violence in past 6 months (n = 246)	Did not experience client-perpetrated violence in past 6 months (n = 843)	Total (n = 1089)	*P*
Socio-demographics				
Age (Mean, SD)	32.6 (9.4)	33.0 (9.8)	32.9(9.7)	
Years of education (Mean, SD)	7.1 (3.2)	7.0 (3.4)	7.1(3.3)	
Married or common-law (vs. other status) (N, %)	72 (29.3%)	225 (26.7%)	297 (27.3%)	
Have children (yes vs. no) (N, %)	218 (88.6%)	734 (87.1%)	952 (87.4%)	
Number of years employed as FSW (Mean, SD)	6.5 (6.6)	6.3 (6.8)	6.4 (6.8)	
Financial need (bad/extremely bad vs. other)(N, %)	81 (32.9%)	191(22.7%)	272 (25.0%)	<0.001
Work context				
Street worker vs. other type of sex worker *(N*,* %)*	81 (32.9%)	197 (23.4%)	278 (25.5%)	<0.01
FSW used alcohol before or during sex with client(s) in past month *(N*, *%)*	170 (69.1%)	491 (58.2%)	661 (60.7%)	<0.01
FSW used drugs^a^ before or during sex with client(s) in past month *(N*, *%)*	31 (12.6%)	45 (5.4%)	76 (7.0%)	<0.001
Total number of clients in past month *(N*, *%)*	46.2 (71.4)	43.8 (54.2)	44.3 (58.5)	
Behavioral & social characteristics				
AUDIT-C Score *(Mean*, *SD)*	7.3 (4.4)	6.8 (4.5)	6.9(4.5)	
Number of drugs^a^ used in the past month *(Mean*, *SD)*	0.23(.60)	0.10 (.37)	0.13(.43)	<0.001
Injected drugs in the past month *(N*, *%)*	42 (17.1%)	70 (8.3%)	112 (10.3%)	<0.001
Community-level factor				
Population of research site municipality				
≤ 499,999 (N, %)	120 (48.8%)	474 (56.2%)	594 (54.5%)	
≥ 500,000 *(N*, *%)*	126 (51.2%)	369 (43.8%)	495 (45.5%)	<0.05

^a^ Mexico’s drug policy reform does not consider drug use illegal unless the amount possessed is above a certain threshold.

### Factors Associated with Client-Perpetrated Violence

#### Factors independently associated with sexual violence

In a multivariate model, seven factors were independently associated with client-perpetrated sexual violence against FSWs (Table 2). Compared to FSWs who did not report client-perpetrated sexual violence in the past six months, those who reported such violence had almost twice the odds of rating their financial situation as “bad” or “extremely bad” (AOR = 2.00; 95% CI 1.31–3.05). FSWs who reported client-perpetrated sexual violence had almost twice the odds of self-identifying as a street worker (vs. other type of sex worker) (AOR = 1.88; 95% CI 1.14, 3.12), twice the odds of having used alcohol before or during sex with clients in the past month (AOR = 2.30; 95% CI 1.33, 3.98), and twice the odds of having used drugs with clients before or during sex in the past month (AOR = 2.19; 95% CI 1.07, 4.49). FSWs who reported client-perpetrated sexual violence had almost three times the odds of having injected drugs in the past month (AOR = 2.92; 95% CI 1.48, 5.75) and had a 32% lower odds of having a higher rating of perceived emotional support from family and friends (AOR = 0.68; 95% CI *0*.48, 0.96). Finally, FSWs who reported sexual violence also had almost twice the odds of living in a city with a population of 500,000 or greater (AOR = 1.87; 95% CI 1.19, 2.94).

**Table 2:** Multivariate logistic regression models examining correlates of sexual, physical, and economic violence against FSWs in 13 sites in Mexico (n = 1089).

	Sexual violence	Physical violence	Economic violence
Socio-demographics	*All values are in the format AOR (95% CI)*		
Age	0.98 (.96, 1.01)	0.99 (.97, 1.01)	1.00 (.98, 1.02)
Marital status (married/ cohabiting vs. other)	1.45 (.95, 2.2)	1.24 (.81, 1.90)	0.92 (.63, 1.33)
Number of years of education	1.01 (.95, 1.07)	1.05 (.99, 1.12)	1.03 (.98, 1.09)
Financial need	2.00 (1.31, 3.05)***	1.74 (1.14, 2.67)**	1.51 (1.04, 2.18)*
Work context			
Street sex worker vs. other	1.88 (1.14, 3.12)*	2.14 (1.27, 3.60)**	1.98 (1.27, 3.09)**
Used alcohol before or during sex with client in past month (y/n)	2.30 (1.33, 3.98)**	2.05 (1.18, 3.56)**	1.50 (.95, 2.36)
Used drugs^a^ before or during sex with client in past month (y/n)	2.19 (1.07, 4.49)*	1.81 (.89, 3.67)	1.83 (.94, 3.56)
Number of clients in past month	1.40 (.90, 2.16)	1.09 (.70, 1.68)	1.01 (.70, 1.45)
Behavioral & social characteristics			
AUDIT-C Score	1.02 (.95, 1.08)	1.04 (.97, 1.11)	1.06 (1.01, 1.12)*
Number of drugs^a^ used in past month	0.61 (.36, 1.05)	3.01 (.38, 23.9)	0.50 (.070, 3.60)
Injected drugs^a^ in the past month (y/n)	2.92 (1.48, 5.75)**	1.45 (.70, 3.03)	1.64 (.85, 3.15)
Emotional support	0.68 (.48, .96)*	0.63 (.44, .89)**	0.72 (.54, .96)*
Community-level factor			
Population of research site municipality	1.87 (1.19, 2.94)**	1.32 (.84, 2.07)	1.20 (.81, 1.76)

AOR, adjusted odds ratio; CI, confidence interval.

*p < 0.05

**p < 0.01

***p < 0.001

^a^ Mexico’s drug policy reform does not consider drug use illegal unless it is above a specific threshold for the amount possessed.

#### Factors independently associated with physical violence

Four factors were independently associated with client-perpetrated physical violence against FSWs in the past six months (Table 2). Compared to FSWs who reported no physical violence during this time frame, FSWs who reported such violence had almost twice the odds of reporting a “bad” or “extremely bad” financial situation (AOR = 1.74; 95% CI 1.14, 2.67), two times the odds of self-identifying as a street sex worker (AOR = 2.14; 95% CI 1.27, 3.60), two times the odds of having used alcohol with clients in the past month (AOR = 2.05; 95% CI 1.18, 3.56), and a 37% lower odds of having a higher rating of perceived emotional support from family and friends (AOR = 0.63; 95% CI *0*.44, 0.89). Three factors that were significant in relation to sexual violence (i.e., use of drugs with clients, injection drug use, population size of city) were not associated with physical violence.

#### Factors independently associated with economic violence

Four factors were independently associated with client-perpetrated economic violence in the past six months (Table 2). Compared to FSWs who reported no economic violence by clients, those who reported such violence had one and a half times the odds of reporting a “bad” or “extremely bad” financial situation (AOR = 1.51; 95% CI 1.04, 2.18), twice the odds of self-identifying as a street worker (AOR = 1.98; 95% CI 1.27, 3.09), 1.1 times the odds of having a higher score on the AUDIT measure of alcohol use (AOR = 1.06; 95% CI 1.01, 1.12), and a 28% lower odds of having a higher rating on perceived emotional support from family and friends (AOR = 0.72; 95% CI 0.54, 0.96).

## Discussion

In this study, which focused on three types of workplace violence perpetrated by FSWs’ male clients in 13 cities throughout Mexico, three correlates emerged as significant in relation to all three types of violence. They were poor financial situation, self-identification as a street worker, and lower levels of perceived emotional support.

FSWs who rated their financial situation as “bad” or “extremely bad” had one and a half to two times the odds of having experienced physical, sexual, or economic violence in the past six months. This finding is consistent with studies of FSWs in other LMIC. In a study in Andhra Pradesh, India, economic hardship (measured by debt, a marker of difficult or dire economic conditions) was associated with almost a three-fold increase in reports of physical violence against FSWs [33]. It has been proposed that women in financial need are more likely to engage in riskier behaviors, such as agreeing to sex in isolated locations, where physical, sexual, and economic violence is more likely to occur [33]. In Mexico, recent structural and economic crises, including unemployment, migration, and poverty, combined with gender inequality and the growing phenomenon of female-headed households, have contributed to increased economic need among women, including FSWs [60]. The association between economic need and violence against FSWs calls for the development of programs to increase economic opportunities for women outside of the sex trade [33].

Self-identification as a street sex worker had the strongest association with all three types of client-perpetrated violence. This finding is consistent with previous studies in developed countries and LMIC [20,33,34,36]. Several studies suggest that street workers are more likely to have sex with clients in isolated venues where there is increased exposure to criminal activity and aggression, including drug use, drug dealing, theft, rape, torture, murder and attempted murder [20,29,61–64]. In Mexico as well as other countries, crimes against FSWs, particularly street workers, are often not reported [36,65,66]. Hence, there is a need for education and prevention programs that address the safety needs of street workers, including strategies for overcoming FSW’s reluctance to report crimes committed against them. Specific changes to laws and policies could also help. In particular, decriminalization of sex work is likely to have a significant impact on prevalence of client-perpetrated sexual violence against FSWs, since women would be more inclined to work in safer indoor environments. Indeed, Shannon et al. [67] estimated that decriminalization of sex work would reduce HIV incidence up to 46% among FSWs and their clients over the next decade.

Low perceived emotional support was associated with all three types of violence against FSWs. In Mexico, poverty and social disadvantage may be so pervasive within some social networks that family members and friends of FSWs who have experienced violence in the workplace are psychologically unavailable to offer support. It is also likely that many FSWs have not disclosed their being a sex worker to family members, thus foreclosing the possibility of this important source of support. High mobility among FSWs may also result in social network members being geographically distant and thus not as available to provide emotional support in times of need. In addition, the long-term effects of prolonged and repeated experiences of violence may lead to feelings of depression and perceptions of low social support [68]. Counseling programs that address longstanding trauma and underlying psychological issues, including low self-esteem, shame, and powerlessness, should be made widely available to FSWs throughout Mexico. Also, community empowerment projects that encourage mutual emotional support among FSWs (e.g., the Sonagachi Project in India) [69] have been found to help women exit the sex trade and lead autonomous lives. Shannon et al. [67] estimated that eliminating client-perpetrated sexual violence against FSWs through the promotion of safer work environments and providing interventions to treat the long-term effects of violence could reduce HIV incidence among FSWs and their clients by up to 20% in the next decade.

Alcohol use by FSWs before or during their sex with clients was associated with about twice the odds of having experienced sexual or physical violence in the past six months, but it was not related to economic violence. There are multiple pathways through which alcohol use before or during sex with clients could influence violence. In several studies, FSWs have reported that among some clients, alcohol use fuels client-perpetrated violence and aggression [23,35,40]. There is also evidence that alcohol use before or during sex with clients is sometimes forced by managers, pimps, bar owners, police and others who may exert control over FSWs [61]. On the other hand, FSWs may voluntarily engage in alcohol use before or during sex with clients as a means of coping with sex work and its negative consequences [39,70,71]. Last, sex workers’ use of substances may negatively influence their ability to make good decisions about their safety [39,40]. The association between alcohol use and client-perpetrated violence indicates an immediate need for prevention programs for FSWs that address the use of alcohol and drugs in the workplace, including helping FSWs find ways to avoid substance use in the work setting, enhancing FSWs’ safety in the context of substance use, providing options for substance use treatment, and increasing FSWs’ access to services to prevent or respond to experiences of violence [20,72]. Some FSWs may also need treatment for clinical depression and PTSD [73], which are known risk factors for substance abuse [14,74,75]. Although government clinics in Mexico currently offer substance use treatment to low-income persons, programs that are specially tailored for FSWs, particularly those with trauma histories, are urgently needed.

Three factors were uniquely associated with sexual violence alone: using drugs before or during sex with client in past month, injection drug use in past month, and population size of city, while the alcohol use score (AUDIT C) was associated only with economic violence. Not surprisingly, the larger a city’s population, the greater the likelihood of sexual violence against FSWs, most likely due to larger cities’ higher crime volume, including sex crimes. The association between population size and crime volume has been found in US studies [76] and is likely to be true in Mexico and other LMIC. The association between drug use before or during sex with clients and sexual violence only could be related to the use of sexually-stimulating drugs, such as methamphetamine or cocaine, that produce sexually compulsive, but not necessarily physically violent behaviors. As in our previous work, we found that injection drug use by FSWs was associated with client-perpetrated sexual violence [42]. This association has significant implications for increasing FSWs’ risk for HIV infection and other STIs. The relationship between higher problem alcohol use by FSWs and economic violence against them may reflect crimes of opportunity in which clients take advantage of FSWs’ inebriation to avoid paying them or to steal their money or property. Further research is needed to determine if these relationships are real or statistical artifacts.

This study has a number of limitations. The sample consisted of volunteers in a sexual risk reduction intervention and thus may not accurately represent the generality of FSWs in each city. In particular, time-location sampling can introduce bias by omitting unidentified venues and by excluding potential participants who either do not visit the identified venues or refuse to be screened for eligibility within them [77,78]. The relatively low levels of violence reported by our sample compared to those in other studies may be due to variations in sampling techniques and definitions of violence [6,79], lack of rapport with interviewers at baseline, or FSWs’ general reticence to report violence that occurs in the context of sex work [33]. The low levels of reported violence may also have resulted in insufficient power to detect associations between some correlates and the outcomes. Further, the use of cross-sectional data precludes the possibility of establishing the temporal direction of associations. This study also lacks data on potentially significant psychological factors (e.g., anxiety, depression) that may have influenced FSWs’ self-reported behaviors. Another limitation was our division of participants into street workers versus “other,” which resulted in the lumping together of call girls with a wide variety of place-based sex workers such as barmaids and brothel workers. This may have obscured important differences in violence experiences among these different types of sex worker, which one might expect because of differences in such contextual factors as workplace support, security, and oversight (or the lack thereof). Last, although all the women in our sample were at high risk for STI/HIV, behavioral risk factors associated with HIV seropositivity (e.g., injection drug use) may increase FSWs’ vulnerability to client-perpetrated violence; thus, future studies on violence should include HIV+ FSWs.

Our findings suggest that approaches to violence prevention for FSWs should be multi-dimensional, potentially involving any or all of the following: addressing barriers (e.g., fear of arrest, substance use, economic hardship) to FSWs’ reporting violence against them [69], teaching strategies to FSWs for reducing the risk of violence in their workplaces (e.g., not using drugs and alcohol with clients, relying on others for protection) [79], delivering anti-violence curricula to clients of FSWs [6,80], decriminalizing sex work [67], and increasing economic opportunities for women outside of the sex trade [33]. There is also an urgent need to train health care workers to recognize signs of sexual and physical violence among FSWs so that appropriate treatment and psychological services are offered in health care clinics throughout Mexico [80].

## Supporting Information

S1 Dataset mx.fsw.violence.v2.sav. File is in SPSS format. (SAV)
